# A Biofeedback-Assisted Stress Management Program for Patients with Irritable Bowel Syndrome: a Randomised Controlled Trial

**DOI:** 10.14806/ej.26.1.980

**Published:** 2021-10-04

**Authors:** Konstantina Exarchopoulou, Anna Papageorgiou, Flora Bacopoulou, Elli Koumantarou Malisiova, Dimitrios Vlachakis, George P. Chrousos, Christina Darviri

**Affiliations:** 1 Postgraduate Course of Science of Stress and Health Promotion, School of Medicine, National and Kapodistrian University of Athens, Athens, Greece; 2 University Research Institute of Maternal and Child Health & Precision Medicine and UNESCO Chair on Adolescent Health Care, National and Kapodistrian University of Athens, Aghia Sophia Children’s Hospital, Athens, Greece; 3 Laboratory of Genetics, Department of Biotechnology, School of Applied Biology and Biotechnology, Agricultural University of Athens, Athens, Greece; 4 Lab of Molecular Endocrinology, Center of Clinical, Experimental Surgery and Translational Research, Biomedical Research Foundation of the Academy of Athens, Athens, Greece

## Abstract

Irritable bowel syndrome (IBS) is a functional gastrointestinal disorder that affects the functionality and quality of life of the affected persons. There is a well-established detrimental reciprocal relationship between stress and IBS. In this randomised controlled trial, IBS patients were randomly assigned to an 8-week biofeedback-assisted stress management intervention group (n=24) or a control group (n=22). Psychometric measures were performed at baseline and following intervention to assess perceived stress, health locus of control and depressive symptoms. In patients of the intervention group, perceived stress and symptoms of depression were significantly decreased, while the sense of control over health was increased. The intervention program was beneficial to the health and quality of life of individuals with IBS. Future randomised controlled studies with larger samples and longer follow-up are required to establish the effectiveness of stress-management techniques in functional gastrointestinal disorders.

## Introduction

Irritable Bowel Syndrome (IBS) is a chronic relapsing-remitting gastrointestinal disorder affecting 10–20% of the population worldwide (Camilleri and Choi, 1997). It is primarily characterised by abdominal pain and abdominal distension, bloating, feeling of incomplete emptying or urgency for defecation and alterations in bowel habits in the absence of structural abnormalities (Guthrie et al., 2002 (Pellissier et al., 2010). In general, although IBS is not a severe disease, it affects the functionality and quality of life of the affected persons (Nelessen *et al.*, 2013).

Possible risk factors for IBS are bacterial gastroenteritis, mucosal inflammation and qualitative or quantitative changes of the intestinal microflora (Tornblom *et al.*, 2007; Trabane *et al.*, 2007; Quigley *et al.*, 2013). Among the well-known triggering factors, stress constitutes a cardinal risk factor for both the IBS onset and relapse. More specifically, stressful life events tend to exacerbate IBS symptomatology in most patients, while traumatic life events (*i.e.* verbal, physical or sexual abuse) are positively correlated with high prevalence of IBS (Palsson and Whitehead, 2013). In addition, people with IBS are more likely to suffer from post-traumatic stress disorder (Drossman et al., 1996) or other co-morbid psychiatric disorders, such as depression or generalised anxiety disorder (Palsson and Whitehead, 2013). People under chronic stress face severe IBS symptoms and have less chance of recovery compared to patients not exposed to stressful events (Bennett et al., 1998). It is worth mentioning that both acute and chronic stress is related to poor adjustment to IBS, showing for example poor compliance to suggested medications (Zernicke *et al.*, 2012). Finally, at least 50% of patients describe depressive symptoms and anxiety, and very often the symptoms can be the result of somatisation and expression of their negative stress (Chang et al., 2011).

IBS is considered as a biopsychosocial disorder (Chang, 2011). The role of brain-gut interactions in the pathogenesis of IBS has been underlined since research has discovered the broad bidirectional communication network between them, known as brain-gut axis (Shah et al., 2020). Sympathetic and parasympathetic pathways of the central nervous system (CNS) interact with enteric nervous system (ENS), regulating the gastrointestinal tract. Both hypothalamus-pituitary axis (HPA) and autonomic nervous system (ANS) over-activity have been recognised to exacerbate IBS symptoms, attesting the mediating role of stress-related neuroendocrine mechanisms on IBS pathophysiology (North et al., 2004). Although few studies have been published on endocrine abnormalities in IBS patients, it has been shown by several studies that alterations in autonomic function can prompt visceral hypersensitivity and changes in gastrointestinal motility (Dobbin et al., 2013; Elsenbruch and Orr, 2001). More specifically, the stress-induced activation of the sympathetic autonomic system signals an increased secretion of catecholamines (*i.e.* epinephrine and norepinephrine) which affects sympathovagal balance and gut homeostasis via neural connections of the brain-gut axis (Brzozowski et al., 2016).

IBS is a benign disease which has been linked to impaired quality of life and high healthcare costs. However, it lacks trustworthy therapeutic options (Lee and Park, 2014). Given that pharmacologic approaches tend to address mainly symptom control, many patients are particularly reluctant to receive symptom alleviating drugs, preferring alternative non-pharmaceutical therapies such as cognitive behavioural therapy, psychotherapy, or even hypnotism (Drossman et al., 2009). Interestingly, such interventions have been showed to be more efficient in reducing IBS than drug therapy (Zernicke *et al.*, 2012). Research has provided evidence regarding the effectiveness of stress management, mindfulness, progressive muscle relaxation, autogenic training and biofeedback training for the improvement of IBS symptoms, quality of life, body pain and overall physical and mental health among IBS patients (Kearney et al., 2011; Heymann-Monnikes et al, 2000; Shinozaki et al., 2010; Patcharatrakul and Gonlachanvit, 2011). Biofeedback training in particular is thought to restore sympathovagal balance leading to symptom improvement (Sowder et al., 2010). Several studies have used biofeedback signals (*e.g.* EMG, HRV, thermal biofeedback etc.) as non-specific relaxation methods in order to alleviate the effects of stress in people with IBS (Blanchard and Schwarz, 1987; Dobbin et al., 2013; Whitehead, 1992). The goal of biofeedback training, in general, is to help patients gain control over biological functions and reactions that are usually unaware of, so that they can self-regulate, decrease sympathetic activity and optimise their health (Chiarioni and Whitehead, 2008).

The aim of this study was to investigate the effect of a biofeedback-assisted stress management program consisting of relaxation breathing (RB) and progressive muscle relaxation (PMR) on physical symptoms and mental health, namely stress and depressive symptoms and health locus of control (HLC), of patients suffering from IBS.

## Materials, Methodologies and Techniques

### Study design

This was a two-armed, parallel group, non-blinded, randomised clinical trial, using balanced (1:1) groups (intervention vs. control). The study was conducted at the outpatient gastroenterology clinic of 417 Army Share Fund Hospital in Athens, over a period of 8 months. The study protocol was approved by the hospital’s Scientific and Ethics Committee and was consistent with the declaration of Helsinki. Patients were informed precisely by the researcher about the study objectives and procedures and were enrolled in the study only after providing written informed consent.

### Participants

The inclusion criteria were: age 18 to 65 years, diagnosis of IBS according to the diagnostic criteria of Rome III, Greek nationality, residency in Athens, and literacy of Greek language. Exclusion criteria were: psychiatric co-morbidity (*i.e.* major depression, psychosis or drug abuse), metastasis or autoimmune disease, systematic corticosteroid intake, previous participation in any study related to stress management, and inability to read or write in the Greek language.

### Randomisation

All outpatients who presented at the gastroenterology clinic and met the study inclusion criteria were randomised into two groups, the control group or the intervention group, based on random numbers generated by an online random generator^1^.

### Intervention

All participants were given written and verbal information about stress and its effect on the onset of symptoms of IBS and quality of life. Psychometric measurements were administered to the participants before the initiation and after the end of the 8-week period. During this period, patients in the intervention group attended five sessions (one every 15 days). Similarly, patients in the control group were contacted every 15 days by the researcher via telephone and asked about their symptoms, mood state and stress. Individuals in the intervention group were administered a CD with instructions for RB and PMR and were instructed to practice them twice per day for 8 weeks. Progressive muscle relaxation is a simple technique, during which patients are guided to throb and progressively relax major muscle groups, starting from the toes up to the facial muscles. This technique was formulated by Jacobson in 1938 and has since proven to significantly reduce stress in healthy subjects and in patients with various diseases (Varvogli and Darviri, 2011). The same patients received biofeedback-assisted stress management training for 15 minutes in every session, focusing mainly on RB. For this purpose, the Nexus-4®a certified by the European Union medical device was used. This is a portable device used to train the patient to monitor the physical reactions (such as respiration rate, quality of a single breath, heart rate and heart rate variability with breathing) in conditions of stress and relaxation, as well as to familiarise the person with stress management techniques by observing the aforementioned physical parameters. After the completion of two months, the control group received the CD with the progressive muscle relaxation and a training session in biofeedback.

### Baseline and Outcome Measures

Socio-demographic and anthropometric variables. Participants were asked about their age, gender, marital status, parenthood, educational level, smoking habits, height, and weight.

#### Health locus of control (HLC).

The Health Locus of Control, on a theoretical level, describes the belief that one’s health depends on internal factors, namely, their own behaviour (internal control center for health) versus other factors such as luck (external control center for health). It is supported by previous research that patients with a chronic disease have a less internal and more external locus of control than healthy adults (Hobbis et al., 2003). The questionnaire consists of 18 formulations (Wallston et al. 1978). Each person is required to answer to what extent he/she agrees with each of these formulations based on a 6-point Likert-type scale (1=strongly disagree, 6=strongly agree). It consists of three subscales, “internal HLC” (HLC1), “external HLC” (HLC2), and “chance HLC” (HLC3). Internal HLC (HLC1) measures the degree to which a person believes to be responsible for his/her health. External HLC (HLC2) measures how much a person believes that other people are responsible for his/her health. And finally, chance HLC (HLC3) represents the extent to which chance determines health. Summing up the responses, for each subscale the score ranged from 6 to 36 points. Higher scores indicate higher strength of each type of faith for health. The instrument has been validated for the Greek population (Varvogli et al., 2011). The internal validity for each subscale was found to be satisfactory for both the initial and final measurements (Cronbach’s alpha: original, HLC1 0.687, HLC2 0.682, and HLC3 0.62, and final, HLC1 0.69, HLC2 0.69, and HLC3 0.56).

#### Perceived Stress Scale (PSS-14).

The Greek Version of PSS was used to evaluate the extent to which people perceive certain situations in life as stressful (Cohen et al., 1983). The questionnaire rates the frequency of feelings and thoughts during the previous month on a 5-point scale Likert-type (from 0 =never to 4= very often). There are seven positive and seven negative items. Scoring is from 0–56, and higher values indicate that the person felt particularly stressed in the previous month. The questionnaire was validated in the Greek language and good psychometric properties were recorded (Andreou et al., 2011). Internal consistency was excellent for both the initial and final measurements (Cronbach’s alpha 0.9 and 0.911, respectively).

#### Beck Depression Inventory (BDI).

Depressive symptoms were measured using the Greek version of BDI which consists of 21 items, which describe specific symptoms (sadness, pessimism, sense of failure, loss of satisfaction, guilt, feelings of punishment, crying, irritability, social withdrawal, loss of libido etc.) and together assess the severity of depressive symptomatology (Beck et al., 1961). The score ranges from 0 to 62 with higher scores indicating patients with more depressive symptoms. The questionnaire has been validated in the Greek language and good psychometric properties were recorded (Donias and Demertzis, 1983). Internal consistency was very good for both the initial and final measurements (Cronbach’s alpha 0.85 and 0.834 respectively).

#### Self-reported Irritable Bowel Syndrome Questionnaire (SIBSQ).

IBS symptoms were assessed using SIBSQ (Endo et al., 2000). The questionnaire includes 14 questions related to abdominal pain, discomfort, frequency of defecation, feeling of incomplete defecation, bloating, feeling of urgent defecation, concern for bowel symptoms and the effect of stress and the meals in the symptoms of the syndrome. The rating scale is based on seven-point scale Likert (1= not at all, 7=severe symptoms present). The questionnaire has not been validated in the Greek language; however, it was translated with the permission given by the authors. Internal consistency was very good to excellent for both the initial and final measurements (Cronbach’s alpha 0.847 and 0.901, respectively).

### Statistical Methods

Interval variables were presented with medians and ranges (minimum and maximum) and categorical with absolute and proportional values. Between-group comparisons were performed with the use of non-parametric Mann-Whitney U-test test for two independent samples and the Pearson’s exact chi-square. Then, the effect of the intervention on dependent variables such as perceived stress (PSS), health locus of control (HLC) and symptoms of the syndrome (IBS), was studied by the differences between the two groups (scores after minus scores before intervention). Effect size for each comparison was calculated according to the formula: r = Z/N^0, 5^ (Z is derived from the Mann-Whitney test and N is the number of patients in our sample). Cut-offs for this effect size were: 0.5, 0.3 and 0.1 for strong, medium and small effect. The level of significance was set at p<0.05 for all analyses. Statistical calculations were performed using SPSS for Windows (version 20.0) statistical software (SPSS Inc., Chicago, IL, USA).

## Results

**Figure 1.**
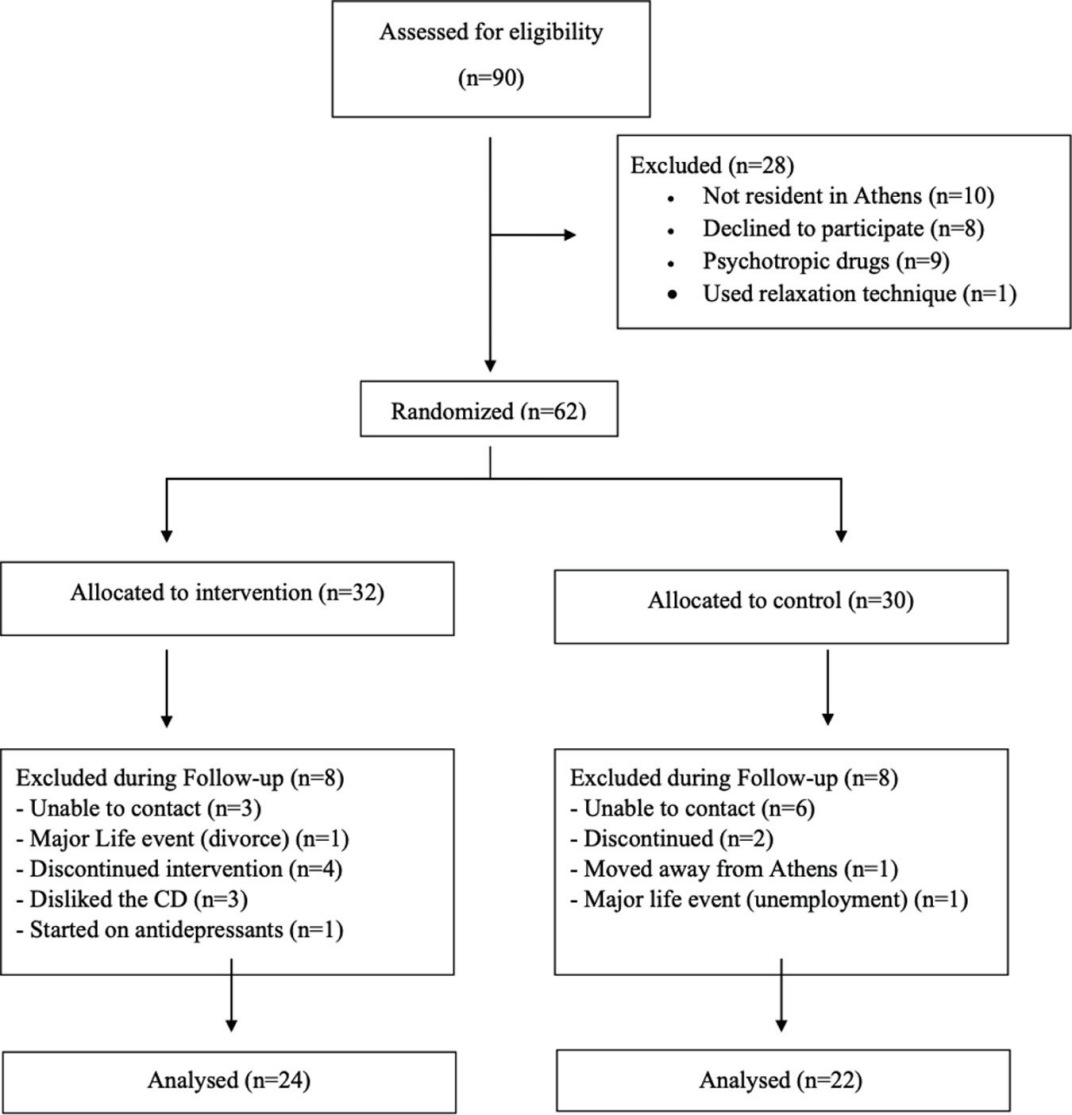
Flowchart of study participants.

The flow diagram of the study is illustrated in Figure 1.

Initially, 90 people were assessed for eligibility, of which 62 entered the process of randomisation, as 8 people refused to participate stating that they did not feel stressed, or did not have time for appointments, or their family did not agree to participate. The remaining 20 subjects were not included according to the exclusion criteria of the study. Specifically, 10 subjects were not residents of Athens, nine were using antidepressants and one subject applied other relaxation techniques such as dance therapy and yoga. Of the 62 people, 32 were randomised to the intervention group and 30 in the control group. During the follow up period, three individuals from the control group and 6 from the intervention group were noted as drop-outs because the researcher was unable to contact them. In addition, three people from the intervention group abandoned the technique because they were not fond of the procedure. One participant of the control group received antidepressants, while a person in the control group relocated away from Athens. Finally, a person in the intervention group got divorced and one person in the control group lost his job. Those two events are major stressful life events, so these individuals were excluded from the analysis. Finally, a total of 46 patients (22 controls and 24 subjects in the intervention group) completed the study and their results were analysed.

### Baseline Analyses

The baseline characteristics of the two groups are presented in Table 1.

According to the results most participants were middle-aged women, married with children, non-smokers, with an average of 16 years of education. The median Body Mass Index (BMI) was higher than 26. There were no significant baseline differences between the two study groups (p> 0.05)

### Primary Endpoint Analyses

Adjusted mean differences, standard deviations, p values, and effect sizes for the intervention group versus the control group for each primary outcome are presented in Table 2.

According to the results, in the intervention group there was a significant (p <0.001) reduction of perceived stress (mean difference ± SD: −6.16 ± 4.30) compared to the control group (mean difference ± SD: 0.50 ± 3.59). According to effect size (0.676), we conclude that the stress management program had a large effect on perceived stress of the patients. Similarly, there was a reduction of symptoms of depression. Finally, the score of symptoms of the syndrome decreased significantly after the intervention for subjects who applied the techniques (difference score = −18.08 ± 8.42), while the control’s group score increased (difference score = 2.68 ± 7.03). The effect size large (0.840), therefore the effect of intervention in symptoms is significant. Additionally, significant variation was observed in the assessment of the control center for health in the control group, as individuals increased the internal control center (difference score = 2.83 ± 2.80) and reduced the external (difference score = −2.12 ± 2.77) and luck (difference score =−2.45 ± 2.39). The difference in the score after the end of the intervention between the two groups is significant, while the effect of the intervention is strong for the external control center and the center of chance, and moderate for the internal health control. Regarding body mass index this significantly decreased in the intervention group compared with the control group (p<0.001).

## Discussion

It is obvious that irritable bowel syndrome is a complex disorder that negatively affects the quality of life and the functionality of patients. The exposure of individuals to chronic or acute stress constitutes a possible risk factor for the syndrome. We conducted this randomised controlled study to assess stress management treatment comprising of an 8-week program, which included training of the participants in relaxing themselves using biofeedback method, discussion of issues related to stress and application of progressive muscle relaxation at home, in a group of patients with irritable bowel syndrome. According to the results of the study, the two groups of the study did not differ on key factors such as age, sex, smoking habits, level of education and the levels of stress, depression, and symptoms of syndromes at the beginning of intervention. After 8 weeks, there was a significant reduction in perceived stress, depressive and irritable bowel syndrome symptomatology in the intervention group. Total scores of these variables differed significantly from the corresponding scores of the control group, while the degree of impact of the intervention on each parameter was strong.

Our results are consistent with previous results showing improvement of symptoms and quality of life in patients receiving a stress management program focused on coping with stress and self-improvement techniques along with drug therapy (Heymann-Monnikes *et al.*, 2000). As reported by studies using cognitive therapy, the levels of stress and depression of our intervention group were reduced, while IBS was improved (Deechakawan et al., 2011; Ljotsson et al., 2011). In a less complex intervention, administration of autogenic training in 21 IBS patients resulted in an additional amelioration of body pain and overall improvement of both physical and mental health (Shonozaki *et al.*, 2010). Similar results have been obtained by using different stress management techniques, such as meditation and mindfulness Relaxation Response Meditation (RRM) (Kearny et al., 2011; Keefer et al., 2002; Asare et al., 2012). With regard to health locus of control (HLC), it has been supported that high levels of external HLC may be a partial mediator of the stress-illness relationship because of the more passive coping strategies enforced by individuals (Artemiadis et al., 2012; Hutner and Locke, 1984). Our study provides further evidence on the reciprocal relationship between control attributions and development and/or exacerbation of IBS (Koloski et al., 2006). Additionally, other studies have highlighted the efficacy of progressive muscular relaxation on the development of a strong internal locus of control (Pawlow and Jones, 2005).

Concerning biofeedback, our results are in line with the results of previous studies using the particular method to relieve patients for IBS symptoms (Leahy et al., 1998; Dobbin et al, 2013; Tremback *et al.*, 2009). However, since no other study has used the exact same combination of relaxation techniques and assessment tools, it is quite precarious to compare these findings to other research.

The association between stress and IBS has been attested by many studies proposing diverse biological pathways (Chang et al., 2011). It is clear, however, that relaxation techniques can have positive effects in the management of IBS. Based on this, we can cautiously conclude that reduction of stress can improve symptoms of the IBS, however we have not found studies to prove the physiological mechanism that explains it. Additionally, it is well known that at least 50 % of patients describe depressive symptoms and stress, IBS is therefore a disorder that is related to the psychology of the individual. In a studyconducted by North and his colleagues in 2004, 25% of women included in the study and a further 5% had somatisation that led to many gastrointestinal and other symptoms. Hence, another interpretation can be based on the effect of the benefits of relaxation techniques to the psychology of individuals (Lakhan et al., 2013).

This study has some limitations. First, our primary outcome measures were based on self-administered self-reports expressing a subjective view of the IBS symptoms as opposed to objective clinical and/or laboratory assessments. Furthermore, there are no validated or clinically meaningful cut-offs for our primary outcomes. Therefore, the translation of our results to everyday clinical practice is impaired. Finally, the small sample size and short duration of the intervention program and follow-up could have influenced the results.

In conclusion, we found that the application of an 8-week stress management program with biofeedback training and other relaxation techniques improved significantly important health and quality of life indicators of individuals with IBS. Namely, it alleviated stress, depressive symptoms and IBS symptoms, and reduced externalising problems in the intervention group. Future randomised controlled studies with larger samples and longer follow-up are required to establish the effectiveness of stress-management techniques in functional gastrointestinal disorders.

## Figures and Tables

**Table 1.** Baseline socio-demographic and disease-related characteristics of study participants.

Main baseline data	Intervention group (24)	Control group (22)	p - value
**Median Age(range)**	49 (24–76)	48.5 (24–85)	0.849
**Sex**			0.746
**-Women**	18 (75%)	15 (68.2%)	
**-Men**	6 (25%)	7 (31.8%)	
**Maternal Status**			0.72
**-Married**	15 (62.5%)	13 (59.1%)	
**-Single**	8 (33.3%)	6 (27.3)	
**-Divorced**	1 (4.2%)	1 (4.5%)	
**Smoking Habits**			0.354
**-Non-smokers**	13 (54.2%)	12 (54.5%)	
**-Smokers**	4 (16.7%)	7 (31.8%)	
**-Former**	7 (29.2%)	3 (13.6%)	
**Children**			0.763
**-Yes**	15 (62.5%)	15 (68.2%)	
**-No**	9 (37.5%)	7 (31.8%)	
**Median BMI (range)**	26.44 (19.53–32.87)	26.92 (20.70–33.64)	0.277
**Median Education age (range)**	16 (12–22)	16 (12–18)	0.063
**Median PSS score (range)**	29.5 (6–54)	27 (9–35)	0.311
**Median HLC1 (range)**	28 (18–36)	27 (15–34)	0.139
**Median HLC2 (range)**	25 (11–34)	25.5 (9–35)	0.935
**Median HLC3 (range)**	17.5 (7–34)	15.5 (7–29)	0.414
**Median BDI score (range)**	11.5 (0–37)	9 (0–27)	0.537
**Median IBS score (range)**	44.5 (19– 68)	45 (17–69)	0.952

PSS= Perceived Stress Scale, HLC= Health Locus of Control (1 = internal, 2= external, 3= chance), BDI= Beck Depression Inventory, IBS.S = Irritable Bowel Syndrome Symptoms, BMI = Body Mass Index (weight/ height2). Tested by the Fisher’s Exact Test chi-square and non-parametric Mann–Whitney U-test. p<0.05.

**Table 2.** Adjusted mean changes of primary outcomes (PSS, BDI, IBS.S) by study group before and after the intervention and effect sizes during the study.

Main baseline data (mean ± SD)	Intervention group (24)	Control group (22)	p - value	Effect size
**APSS_score**	−6.16±4.30	0.50±3.59	<0.001	0.676
**ΔBDI_score**	−3.75±3.60	0.22±1.50	<0.001	0.687
**ΔIBS.S_score**	−18.08±8.42	2.68±7.03	<0.001	0.840
**ΔHLC1**	2.83±2.80	0.86±1.48	0.007	0.394
**ΔHLC2**	−2.12±2.77	0±2.20	<0.001	0.515
**ΔHLC3**	−2.45±2.39	−0.27±1.35	<0.001	0.629
**ΔBMI**	−0.48±0.71	0.11±0.31	<0.001	0.531

Notes: PSS= Perceived Stress Scale, BDI= Beck Depression Inventory, IBS.S = Irritable Bowel Syndrome Symptoms, HLC = Health Locus of Control (1= internal, 2= external, 3= chance), BMI= weight/ height2. Tested by the non-parametric Mann–Whitney U-test. p<0.05.

## Key Points

- There is a well-established reciprocal relationship between stress and irritable bowel syndrome (IBS).
- 46 IBS patients were randomly assigned to an 8-week biofeedback-assisted stress management intervention group or a control group.
- In patients of the intervention group, perceived stress and symptoms of depression were significantly alleviated, while the sense of control over health was increased.

## Footnotes

1
www.random.org
